# Influence of Appendicitis on Hepatic Insufficiency

**Published:** 1913-11

**Authors:** 


Influence of Appendicitis on Hepatic Insufficiency. M. J. Duvergey, *Paris Med.,* May 10, 1913. In grave infections there is diminished excretion of urea, followed by excess during convalescence; in subacute cases, there is a constant excess; in chronic cases no modification. Alimentary glycosuria and urobilinuria are noted in appendicitis of marked degree. The association of diminished urea, alimentary glycosuria and urobilinuria is a grave omen. If the curve of urea elimination is followed, one has a check on the course of the disease.

